# Kinetic and thermodynamic characterisation of HIV-protease inhibitors against E35D↑G↑S mutant in the South African HIV-1 subtype C protease

**DOI:** 10.1080/14756366.2019.1636234

**Published:** 2019-08-14

**Authors:** Sibusiso Maseko, Eden Padayachee, Siyabonga Maphumulo, Thavendran Govender, Yasien Sayed, Glenn Maguire, Johnson Lin, Tricia Naicker, Sooraj Baijnath, Kruger Hendrik Gerhardus

**Affiliations:** a Catalysis and Peptide Research Unit, School of Health Sciences, University of KwaZulu-Natal, Durban, South Africa;; b Protein Structure-Function Research Unit, School of Molecular and Cell Biology, University of the Witwatersrand, Wits, South Africa;; c School of Chemistry and Physics, University of KwaZulu-Natal, Durban, South Africa;; d School of Life Sciences, University of KwaZulu-Natal, Durban, South Africa

**Keywords:** Protease, HIV, mutant, inhibitor, thermodynamics

## Abstract

Herein, we report the effect of nine FDA approved protease inhibitor drugs against a new HIV-1 subtype C mutant protease, E35D↑G↑S. The mutant has five mutations, E35D, two insertions, position 36 (G and S), and D60E. Kinetics, inhibition constants, vitality, Gibbs free binding energies are reported. The variant showed a decreased affinity for substrate and low catalytic efficiency compared to the wild type. There was a significant decrease in the binding of seven FDA approved protease inhibitors against the mutant (*p* < .0001). Amprenavir and ritonavir showed the least decrease, but still significant reduced activity in comparison to the wildtype (4 and 5 folds, respectively, *p* = .0021 and .003, respectively). Nelfinavir and atazanavir were the worst inhibitors against the variant as seen from the IC_50_, with values of 1401 ± 3.0 and 685 ± 3.0 nM, respectively. Thermodynamics data showed less favourable Gibbs free binding energies for the protease inhibitors to the mutant.

## Introduction

The human immunodeficiency virus (HIV) is a retrovirus from the Retroviride family and is responsible for acquired immune deficiency syndrome (AIDS), which was first reported in 1981^1^. This infection is controlled by the use of antiviral drugs which helps reduce the mortality and morbidity as well as promote increased patient life expectancy^2^.

Protease inhibitors (PIs) are one class of antiviral drugs that target an essential viral enzyme, HIV-1 protease^3–5^. The role of HIV-1 protease in the processing of Gag and Gag-Pro-Pol polyproteins into building blocks for individual proteins essential for viral maturation, has made it one of the major targets for drug development^6^. There are currently nine FDA approved protease inhibitors^7^, originally designed for type B HIV PR^8^. These inhibitors represent the most potent anti-AIDS drug reported to date and are essential components of the highly active antiretroviral therapy (HAART)^9^. HAART is credited with significantly lowering AIDS-related deaths, and is currently implemented to the whole world as the standard care for HIV-AIDS treatment^9^.

The emergence of drug resistant mutants in the HIV-PR has become a huge problem with the increased failure of HAART^10^^,^^11^. Newly infected patients are infected with resistant strains, which are an added challenge in the treatment of HIV infections^12^. Herein we report the effect of a variant protease in the South African HIV-1 subtype C PR on the binding capacity of the nine commonly used PIs. The variant has the following mutations; E35D, I36G, two insertions at position 35 (G and S), and D60E and is referred to as E35D↑G↑S (Figure S1) with the upward arrows showing positions of insertions^13^. The variant was discovered in a HIV-1 positive mother who participated in a PMTCT (Prevention of Mother-To-Child Transmission) cohort. This mutant was discovered from different patient reported by Lockhat et al. and Maseko et al. ^14^^,^^15^. This mutant is also different from the I36T↑T mutant as bears to insertions compared to just one from the former. The patient received treatment with the following reverse transcriptase inhibitors (RTIs): efavirenz, d4t (stavudine), and 3TC (lamivudine). Interestingly, the patient was completely drug-naïve with respect to protease inhibitors^14^.

**Figure 1.**
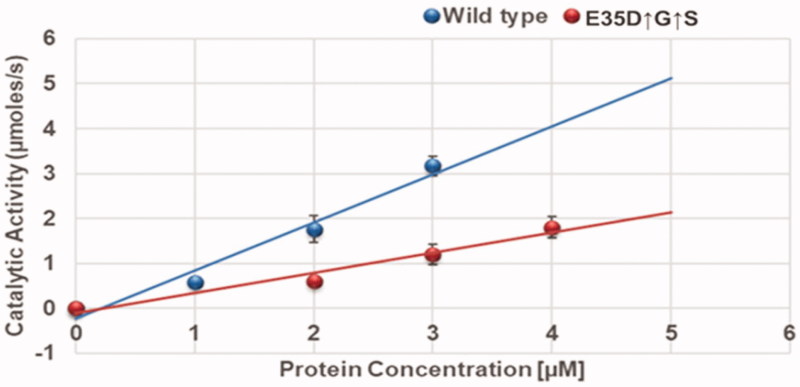
Determination of enzyme turn-over number. Linear curves for determining the turn-over number (*k_cat_*) of the wild-type and the variant. Turn-over number was determined from the slopes of the plots. The experiments were performed at 37 °C in 10 mM sodium acetate buffer, 0.1 M sodium chloride, pH 5.0, at a substrate concentration of 250 µM. The experiments were conducted in triplicates and the data are reported as the mean ± SD. (*n* = 3).

The variant protease together with the wild type C-SA HIV protease were cloned, purified, and characterised^13^. The purified variant protease possessed weaker catalytic activity compared the wild type^13^.

## Materials and method

### Protein expression and purification

Protein expression was performed as reported previously^13^. Briefly, C-SA HIV protease and mutant E35D↑G↑S were cloned in pGEX-6P-1 and expressed in *Escherichia coli* BL21 (DE3) pLysS. Cells were harvested 4 h after IPTG induction by centrifugation (8000*× g*). Cells were then resuspended in ice-cold buffer A (10 mM Tris HCl, 5 mM EDTA, 1 mM PMSF) ruptured by sonication and lysate was spun at 14,000× *g*. Pellet was washed with buffer A with 1% Triton and again spun at the same speed for 20 min. Pellet containing inclusion bodies was resuspended in buffer B (10 mM Tris-HCl, 5 mM EDTA, 8 M urea, 5 mM DTT) and kept at room temperature for 1 h. Presence of GST tagged protein was verified by SDS-PAGE and western blot using GST antibodies. Protein purification was carried out using AKTA 100–950 (GE Health Care). Partial purification was carried out using a Hitrap QFF cation exchange column (5 ml GE Health care) and the protein of interest was eluted using a NaCl gradient (0–1 M NaCl). Eluted samples were then desalted with using a Hitrap desalting column. Further purification was then carried using a GSTrap affinity column. GST tag was then removed by digestion with preScission protease overnight at 4 °C. All contents were then loaded back on a GSTrap affinity column and HIV protease was collected in the flow through, refolded and stored at −70 °C until further use. The purified proteases were confirmed by SDS-PAGE, Western blot and LC-MS-TOF (Central Analytical Facility, University of Stellenbosch).

### Kinetic parameters

Enzymatic activity of the HIV-1 C-SA and mutant (E35D↑G↑S) protease was measured by following the hydrolysis of the HIV-PR chromogenic substrate, Lys-Ala-Arg-Val-Nle-nPhe-Glu-Ala-Nle-NH_2_ as reported before^13^^,^^15^. The substrate resembles the conserved protease cleavage site, KARVL/AEAM^8^ between the capsid protein and the nucleocapsid p2 in the Gag-polyprotein precursor. Hydrolysis of the HIV chromogenic substrate was characterised by the decrease in absorbance at 300 nm. Catalytic properties such as the K_m_, *k_cat_*, and *k_cat_*/K_m_ of the proteases were determined^8^. All catalytic activity assays were performed using a Jasco V-630 spectrophotometer (Jasco International co., LTD, Japan). The mutant had shown weaker affinity to the substrate on our previous study, therefore the same trend was expected of the inhibitors.

### Inhibition studies

Inhibition constants, K_i_, for the inhibitors (Amprenavir, APV; Atazanavir, ATV; Darunavir, DRV, Indinavir, IDV; Nelfinavir, NFV; Lopinavir, LPV; Ritonavir, RTV; Saquinavir, SQV; Tipranavir, TPV) against E35D↑G↑S were obtained at 37 °C. This was done by monitoring the rate of chromogenic substrate hydrolysis using 2 µM protease in 50.0 mM sodium acetate, 0.1 M NaCl, pH 5.0, and (0–250 µM) substrate in increasing amounts of inhibitor (0–10 nM).

### Vitality

For comparing the relative selective advantage of a given protease mutant over the wild type in the presence of an inhibitor, the catalytic efficiency of the mutant must be included in the calculations. This is done by introducing the term “vitality” which is a measure of resistance. Vitality, v, is defined as v = (K_i_ × K_cat_/K_m_)_MUT_/(K_i_ × K_cat_/K_m_)_WT_, and predicts the therapeutic effect of a given protease inhibitor.

### Fluorescence quenching

Quenching experiments were performed according to the method reported by Maseko et al.^15^. Spectrofluorimetry was used to determine structural changes induced in HIV protease by the interaction of the inhibitors with the purified enzymes using Jasco V-630 spectrofluorimeter (Jasco International co., LTD, Japan). The excitation wavelength was fixed at 295 nm, the wavelength at which tryptophan absorbs and the emission wavelength measured was at 482 nm. The change in fluorescence of a solution was monitored over 10 min, as increasing concentrations of inhibitors were added to a reaction mixture of HIV protease in 50 mM sodium acetate, 1 M NaCl, pH 5 in a final volume of 100 µ. All fluorescence quenching experiments were performed at 4 different temperatures (293 K, 298 K, 303 K, 310 K). The following equations are applicable^16^.

(1) $$F_{0}/F=1+K_{\text{sv}}Q$$

(2) $${\text{lnK}}_{\text{sv}}=-(\Delta H/\text{RT})+(\Delta S/R)$$

where F_0_ and F are the florescence in the absence and presence of quencher, K_sv_ is the Stern Volmer constant, Q is the quencher (drug), ΔH is the enthalpy, ΔS is entropy, R is the gas constant and T is the experimental temperature. The equation is developed from Van’t Hoff relationship^17^.

(3) $$\Delta G=\text{RT}\ln K_{i}$$

### Homology modelling and molecular docking

The crystal structure of the wild-type C-SA HIV protease was obtained from the Protein Data Bank (PDB). The PDB entry for the crystal structure is 3U71 with the C-SA protease complexed with ATV. The homology structure prediction of E35D↑G↑S variant was generated using SWISS-MODEL^18^. The model prediction of the E35D↑G↑S variant was made using the PDB ID 3U71 structure of the South African wild-type HIV-1 subtype-C as a template and this was based on the highest sequence identity of 95.96%.

Molecular docking was performed using the AutoDock software^19^ in order to ensure that the inhibitors are in an accurate orientation in the active site of E35D↑G↑S mutant relative to the wild-type. The Lamarckian generic algorithm was used, and the active site was defined by utilising Auto Grid. The grid box was set as 60 × 60 × 60 points with the spacing set as default 0.375 Å. The 3-D structure of the enzyme complexed to ATV (Figure 5(B)) is available as a PDB format in the Supplementary Material.

### Statistical analysis

Experiments were done in triplicates and results were presented as the mean ± standard deviation. Significance was set to 0.05 and data used were analysed using unpaired *t*-test. GraphPad Prism 7 software was used^18^.

## Results

### Kinetic parameters

The kinetic Michaelis constant was found to be 128.6 ± 1.0 and 198.0 ± 1.0 µM for the wild type (C-SA) and the mutant (E35D↑G↑S), respectively. The lower K_m_ for the wild type means that the wildtype exhibited a higher affinity for the substrate than the mutant. The turnover numbers were determined from the slopes from Figure 1 and were found to be 1.067 ± 0.003s^−1^ and 0.446 ± 0.001s^−1^ for the wildtype and the mutant, respectively. Again, the wildtype exhibits an increased catalytic efficiency (*k_cat_*/K_m_) compared to the mutant. This can be attributed by the fact that K_m_ for the wild type was lower than that of the mutant.

### Inhibition studies

**Figure 2.**
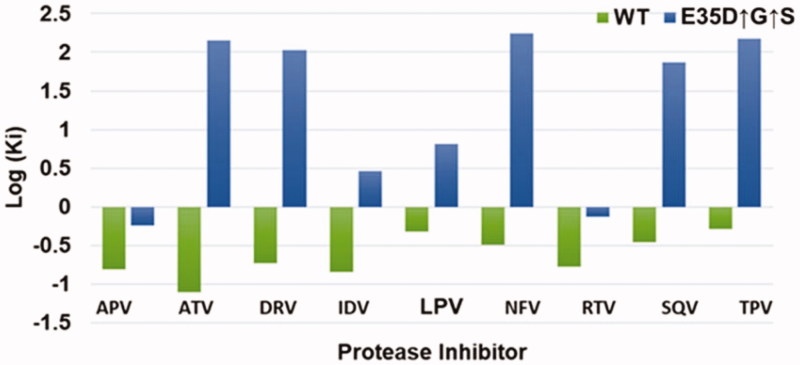
Inhibition constants of the wild type C-SA and the mutant in a logarithmic scale. The wild type (C-SA) is shown in green, and mutant (E35D↑G↑S) in blue. (*n* = 3).

A summary of the inhibition by the nine FDA approved PIs is shown in Figure 2. The figure shows the logarithmic K_i_ values for both the wild type and the mutant. For the wild type, all the inhibitors exhibited negative log K_i_ values meaning all the inhibitors had K_i_ values less than 1.0 nM. ATV was the best inhibitor (log K_i_ = −1.11) against the wild type as seen from Figure 2. TPV was the poorest inhibitor (log K_i_= −0.29) against the wild type. Overall, all nine inhibitors are effective against the wild type. For the mutant, only two inhibitors (APV and RTV) exhibited negative log K_i_ values. The other seven inhibitors showed weaker binding to the mutant, and the log K_i_ values of these seven inhibitors were positive. ATV, which showed the best inhibition (log K_i_ value of −1.11) against the wild type and yet the second weakest drug against the mutant with a log K_i_ value of 2.16. NFV was the worst inhibitor against the mutant with a log K_i_ value of 2.24. A graphical example of K_i_ determination is shown in Figure S2.

The IC_50_ values for the two enzymes are summarised in Table 1. IC_50_ values for the wild type are all better than 4.0 nM. This means that all the drugs are much more effective against the wild type than against the mutant. For the mutant APV and RTV were the only effective inhibitors with reasonable IC_50_ values (4.1 ± 0.3 nM and 7.1 ± 0.4 nM, respectively). All the other inhibitors exhibited IC_50_ values above 30 nM. Again, ATV and NFV were the worst inhibitors against the mutant with IC_50_ values of 685.0 ± 3.0 and 1401.0 ± 3.0 nM, respectively. This data agrees with the K_i_ data.

**Table 1.** A summary of IC_50_ values for the wild type C-SA protease and the mutant (*n* = 3)

Inhibitor	WT IC_50_ (nM)	E35D↑G↑S. IC_50_ (nM)
APV	0.81 ± 0.04	4.1 ± 0.05
ATV	1.07 ± 0.08	685.0 ± 3.0
DRV	1.07 ± 0.04	154.10 ± 2.0
IDV	3.44 ± 0.06	33.56 ± 1.0
LPV	2.99 ± 0.01	51.67 ± 0.8
NFV	1.99 ± 0.05	1401.0 ± 3.0
RTV	0.78 ± 0.01	7.1 ± 0.40
SQV	0.77 ± 0.05	114.10 ± 1.2
TPV	1.55 ± 0.02	202.70 ± 2.6

### Vitality

**Figure 3.**
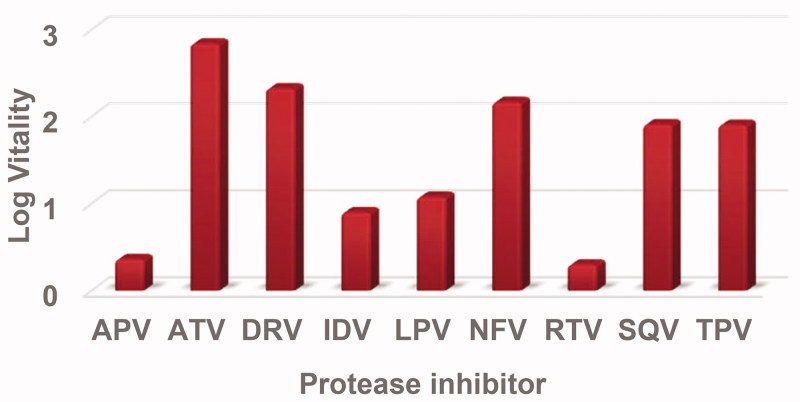
Log vitality values for the E35D↑G↑S mutant protease with respect to the nine inhibitors using wild type as a reference.

Log vitality values are shown in Figure 3 for the E35D↑G↑S mutant protease with the corresponding inhibitor using the wild type as a reference enzyme. The E35D↑G↑S mutant showed low vitality values for APV and RTV displaying log vitality values of 0.36 and 0.30, respectively. IDV and LPV had log vitality values close to 1.00. ATV showed the highest vitality value, meaning the mutant was resistant against ATV. DRV, SQV, NFV, and TPV with all had log vitality values around two.

### Quenching and thermodynamics

**Figure 4.**
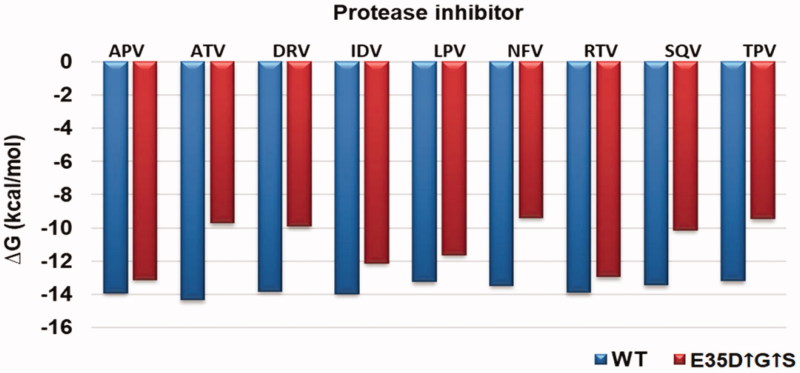
Gibbs free binding free energy of the E35D↑G↑S protease and the wild type C-SA HIV protease. The wild type is represented in blue whilst the E35D↑G↑S is in red.

Thermodynamic parameters were calculated from the Stern Volmer and Van’t Hoff plots (Figures S3 and S4). From these plots, ΔH and ΔS values for each drug were calculated. ΔG values were calculated from the K_i_ values from Equation (3). A graphical presentation of the ΔG values for both the wild type and the mutant is shown in Figure 4. For the wild type, ATV was the best drug with a ΔG value of −14.35 kcal/mol. TPV displayed slightly weaker binding to the wild type with a ΔG value of −13.19 kcal/mol. All the thermodynamic reactions were entropy driven as judged from the big negative (favourable) values. ΔH was positive (unfavourable) for all the inhibitors except for TPV.

For the variant, APV and RTV showed the best binding with **Δ**G values of −13.12 and −12.96 kcal/mol, respectively. The seven other inhibitors showed weaker binding to the mutant as seen from Figure 4. Again, all reactions were entropy driven. **Δ**H values were also positive for the mutant except for TPV.

To compare the effect of the mutations in the binding of PIs with the wildtype ΔΔG, ΔΔH, and − TΔΔS values were calculated and are presented in Table 2. The values are the difference between the wild type and the mutant (mutant-wild type). From the table, it is seen that there was an overall decrease in the binding energies against the mutant. APV and RTV showed less change in the binding with ΔΔG values of 0.80 and 0.93 kcal/mol, respectively. NFV and TPV showed the most decrease in binding with ΔΔG values of 4.63 and 4.10, respectively. There was no observable trend in the ΔΔH and − TΔΔS values. The K_i_ ratios in Table 2 show that overall the nine drugs showed weaker binding to the mutant.

**Table 2.** A comparison of the difference (mutant-wildtype) between the inhibition constants and thermodynamic parameters of the wild type (C-SA) and the mutant (E35D↑G↑S).

Inhibitor	K_i_ ratio	ΔΔG	ΔΔH	−TΔΔS
APV	4 ± 0.1	0.80 ± 0.1	12.9 ± 0.8	12.1 ± 0.2
ATV	1828 ± 2.0	4.6 ± 0.2	3.1 ± 0.1	−1.5 ± 0.1
DRV	560 ± 3.0	3.9 ± 0.5	2.1 ± 0.0	−1.8 ± 0.1
IDV	20 ± 0.5	1.9 ± 0.6	−0.1 ± 0.0	1.8 ± 0.3
LPV	12 ± 0.1	1.6 ± 0.3	−13.1 ± 0.9	14.7 ± 0.1
NFV	532 ± 2	4.1 ± 0.2	2.3 ± 0.0	−1.8 ± 0.0
RTV	5 ± 0.4	0.9 ± 0.1	6.8 ± 0.1	5.8 ± 0.2
SQV	215 ± 2.0	3.3 ± 0.5	2.9 ± 0.1	0.4 ± 0.0
TPV	292 ± 4.0	3.7 ± 0.1	3.2 ± 0.2	−7.1 ± 0.1

### Homology modelling and molecular docking

**Figure 5.**
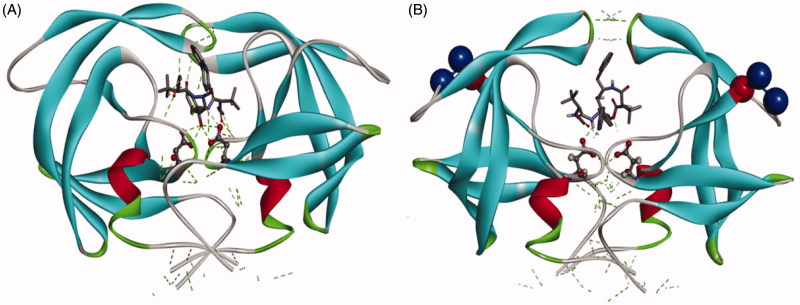
A ribbon representation of the wild type C-SA HIV protease (A) from a crystal structure (3U71) in its homodimeric form and E35D↑G↑S variant (B). The inhibitor complexed to these structures is ATV. Aspartic residues are shown in purple (Asp 25/25′). The mutation (E35D) is in red and the insertions are shown in blue. The figures were created using Discovery Studio 4.0 Visualizer^20^. The docked structure of ATV with the mutant is available as PDB format in the Supplementary Material.

A simple molecular docking diagram showing the interaction of ATV with both the wildtype and the E35D↑G↑S variant is shown in Figure 5.

The mutant showed weaker interaction with the inhibitor. From the figure, it is also seen that the volume of the enzyme binding pocket increased in the mutant. It is possible that a more closed conformation for B will be formed upon molecular dynamics simulation.

## Discussion

The hinge region (residues, 35–42 and 57–61) of the HIV-1 protease is closely associated with the stability and movement of the flap region^21^. The flap region undergoes substantial movement allowing for substrate/inhibitor (open conformation) and forms key interactions during binding of substrate/inhibitor (closed conformation)^22^. The flaps are required to display flexibility. However, increased flexibility may reduce substrate processing and binding of PIs. From the kinetic data (Figure S1), it was observed that the variant had a low affinity for substrate and reduced catalytic efficiency. This observation is in contrast with what was observed in the I36T↑T mutant we recently reported^15^. The I36T↑T mutant showed an increased affinity for substrate and increased catalytic activity^15^. Both mutants (I36T↑T and E35D↑G↑S) are in the hinge region but they show different properties. E35 in HIV-PR maintains long-range interactions within the PR polypeptide chain^23^. A study by Naicker et al. showed that the E35-R57 salt bridge (ion pair) is absent in both monomers of the C-SA HIV PR^21^. They further showed that the R57 in C-SA PR adopts a different rotamer to that of R57 in the subtype B PRs, resulting in the absence of a salt bridge. Interestingly the mutant studied here, also experience a E35D mutation, suggesting that it also does not have a E35-R57 salt bridge. The salt bridge controls movement and decreases flexibility of the flaps^23^. The E35D mutation was reported to induce reduced binding affinities to PIs^8^^,^^21^. The mutant being studied here showed a reduced affinity for the nine FDA approved PIs as seen from the K_i_ and thermodynamic data. Interestingly the wild type was still susceptible to the PIs. The behaviour of the mutant could be caused by the E35D mutation. The mutant in this study though having different properties when compared to the previously reported mutant (I36T↑T)^15^, they both showed weaker binding to PIs.

M36I (present in the wild type) is reported to regulate the size of the binding cavity of the protease and influence the shape of the active site^24^. This mutation is related to NFV and other PIs by complementing other mutations^24^^,^^25^. The wild type contains only the M36I polymorphism. The 36th residue interacts with residues located near the active site^24^. Mutations in this position result in the change in conformation of the binding pocket^24^. This polymorphism does not cause resistance on its own. This explains why the drugs were still effective against the wild type. Binding of the nine protease inhibitors to the wild type in this study was in the same range for reported other non-C HIV-1 proteases. The variant we are studying contain an I36G mutation and showed weaker binding to NFV and had the worst IC_50_ result (Table 2 and Figure 4) with respect to this drug. Overall, the mutant showed reduced binding to all the nine drugs. We proposed that I36G together with the other mutations in E35D↑G↑S caused the reduced binding energies of the PIs.

## Conclusion

The effect of the insertions and mutations in the C-SA HIV-PR was studied. The mutant (E35D↑G↑S) showed decreased affinity for the substrate. Binding to the nine FDA approved inhibitors was also significantly reduced for the mutant. APV and RTV can still be prescribed for patients with this mutant as their IC_50_ values are less 10 nM. The other seven drugs are much less effective. Further studies need to be done to further explain why the variant exhibit reduced binding affinities.

## Supplementary Material

Supplemental Material
