# Supplementary material for: Kinetic and thermodynamic characterisation of HIV-protease inhibitors against E35D↑G↑S mutant in the South African HIV-1 subtype C protease
Source: J Enzyme Inhib Med Chem. 2019 Aug 14;34(1):1451–6. doi: 10.1080/14756366.2019.1636234 (PMC6713120; doi:10.1080/14756366.2019.1636234)
Supplement: Supplemental Material [file IENZ_A_1636234_SM7839.docx]

**Supplementary**

**Kinetic and Thermodynamic Characterization of HIV-Protease inhibitors against E35D↑G↑Smutant in the South Africa HIV-1 Subtype C Protease.**

**Sibusiso B Maseko^a^**, Eden Padayachee^a^, Thavendran Govender^a^, Yasien Sayed^b^,Glenn EM Maguire^ac^, Johnson Lin^d^ ,Hendrik G Kruger^a*^


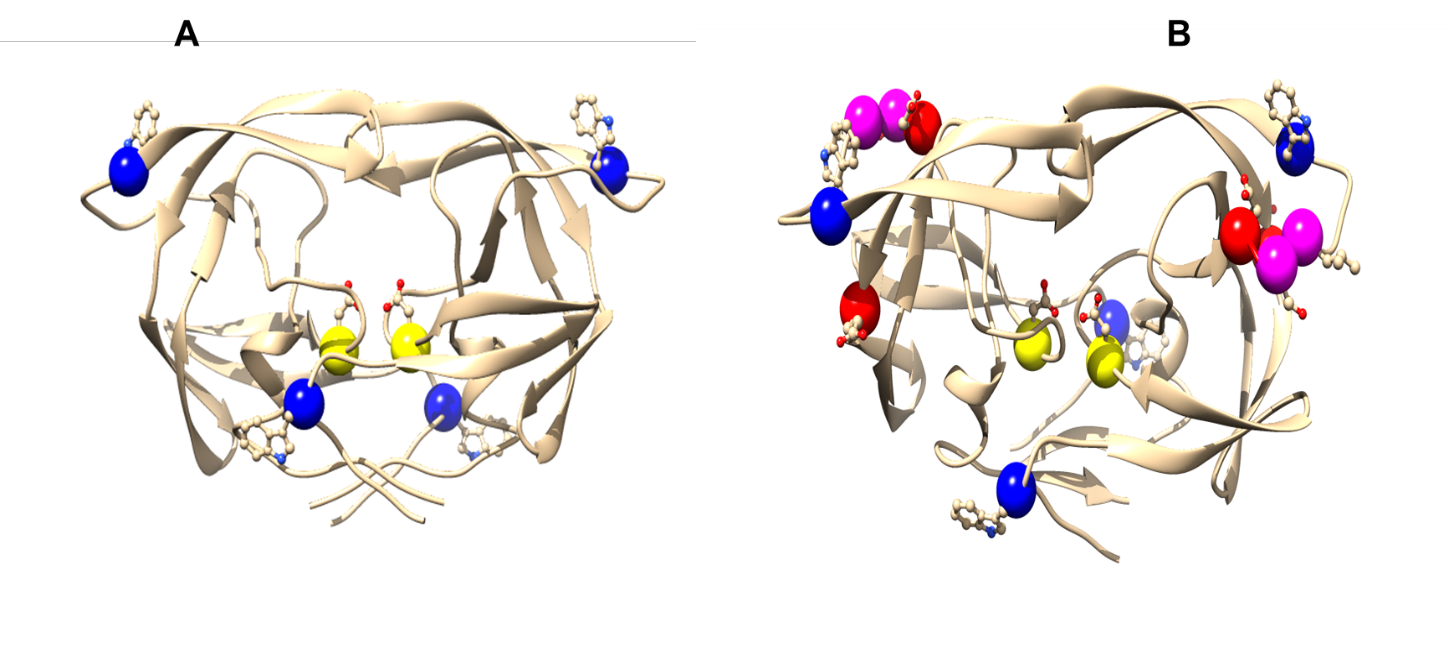


Figure S1. A ribbon representation of the wild type C-SA HIV protease (A) and E35D↑G↑Svariant (B). Shown in yellow is the aspartic residues (Asp 25/25'). The insertions are shown in pink and tryptophan in blue. The red spheres are other amino acid mutations found in this variant protease. The figures were created using UCSF Chimera version 1.9 [1].


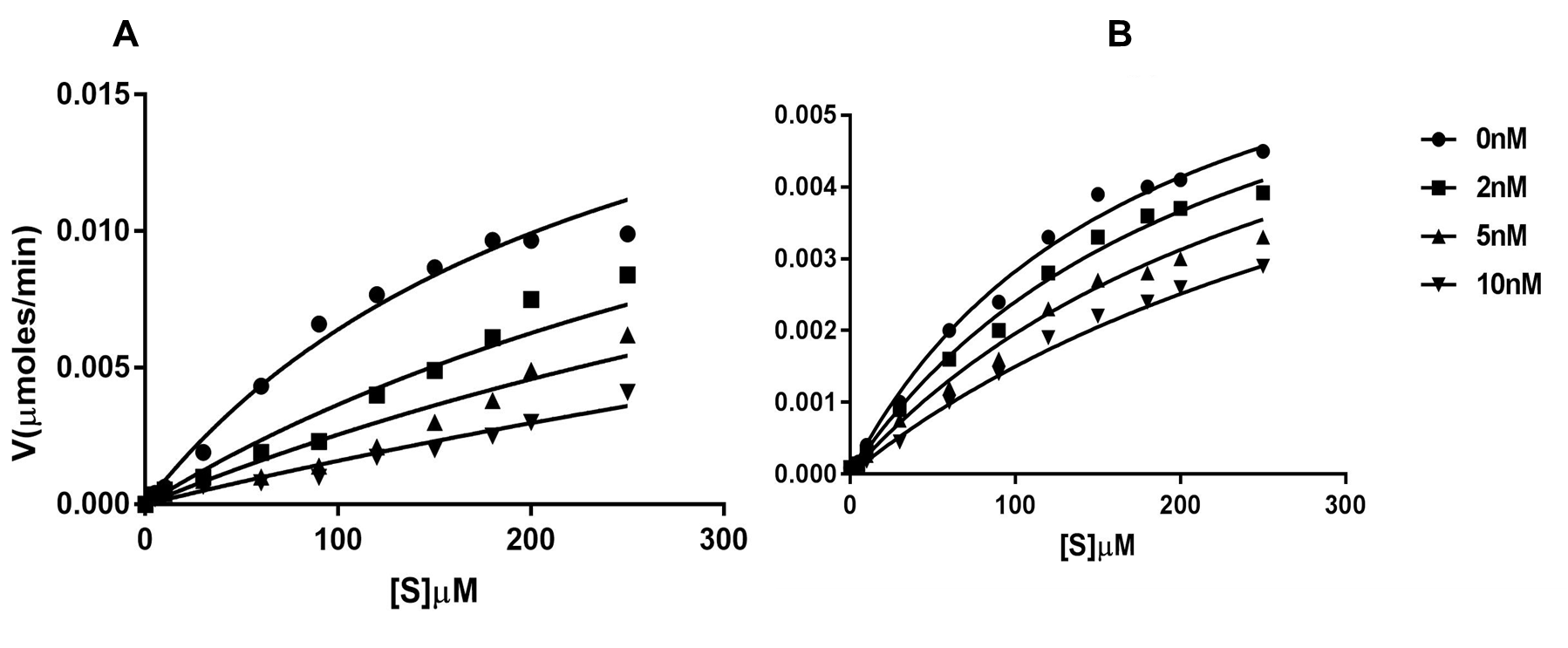


Figure S2: Inhibition (K_i_) of the protease activities of wild type[A] and E35D↑G↑S[B] by RTV The reaction mix contained an increasing substrate concentration (0-250 μM); Protease enzyme (50 nM), protease inhibitor (0-10 nM) in a 96-well plate with a total volume of 100 µL (n = 3).

The K_i_ values were estimated using a competitive inhibition equation (Equation 1) according to Williams et al. [2].

**V =** $\frac{\mathbf{Vmax[S]}}{\boldsymbol{K}\mathbf{m}\left( \mathbf{1+}\frac{\left[ \mathbf{I} \right]}{\boldsymbol{Ki}} \right)\mathbf{+[S}\boldsymbol{]}}$ **(Equation S1)**

[I] is the inhibitor concentration, K_m_ is the Michaelis constant, K_i_ is the inhibition constant, V and V_max_ are the velocity and the maximum velocity of the enzyme, respectively.


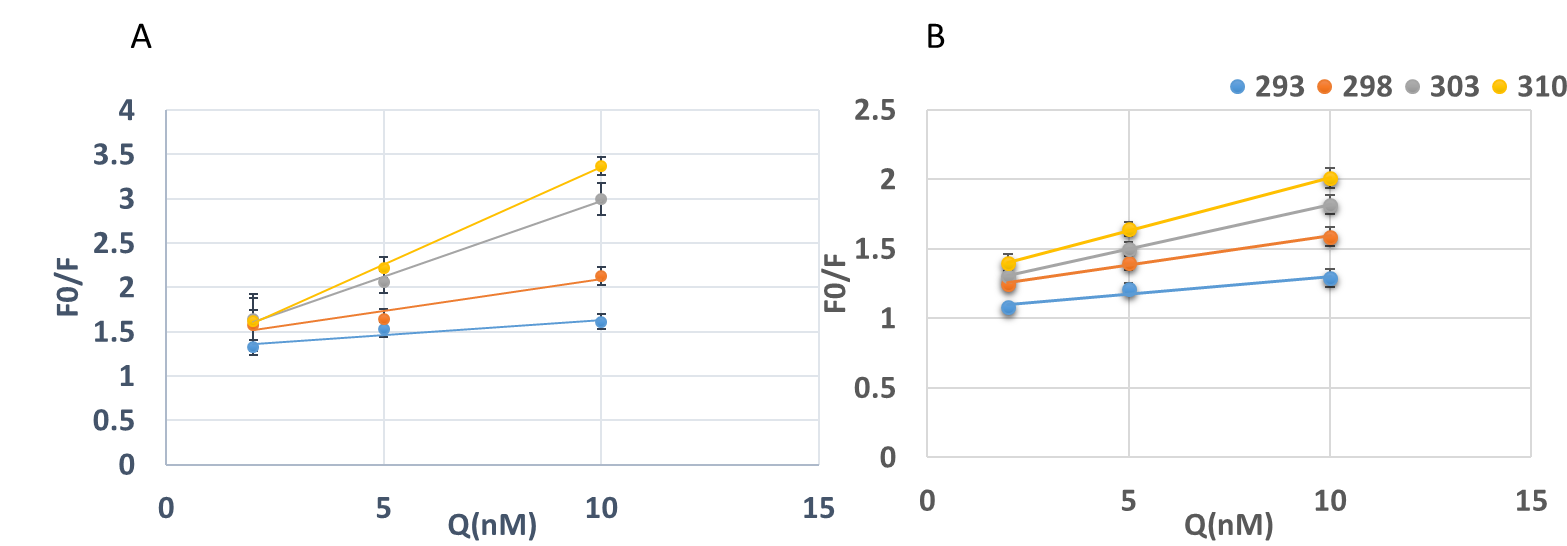


Figure S3: Examples of Stern Volmer plots for fluorescence quenching of WT (A) and the mutant E35D↑G↑Sin 50 mM Sodium Acetate buffer (pH 5) containing NaCl (1M) in a final volume (100 µl) when treated with Ritonavir at different temperatures (n = 3).


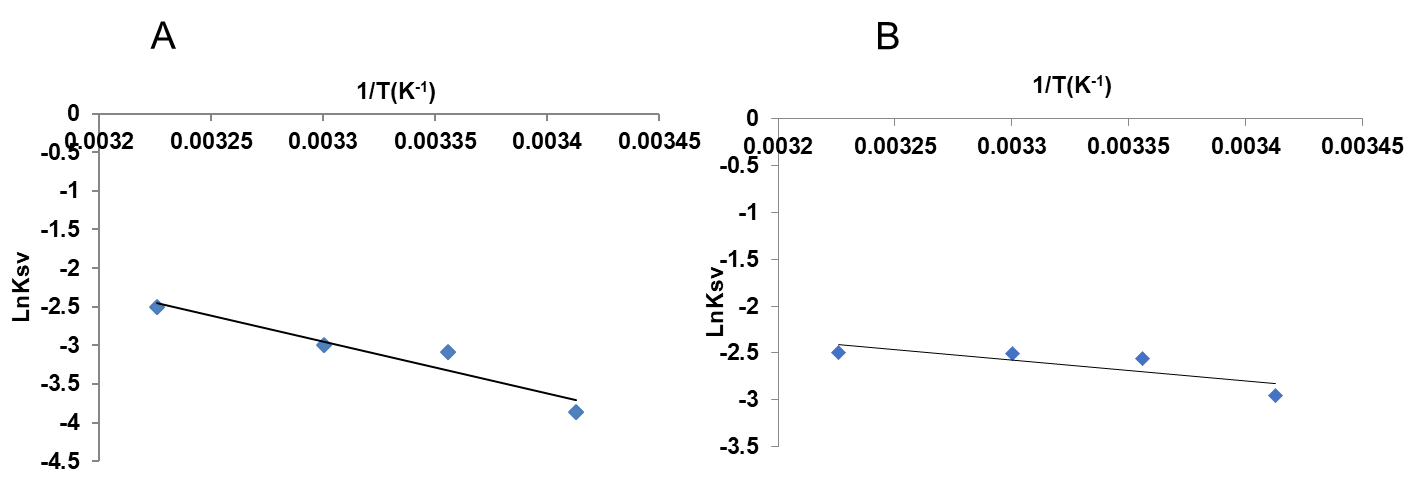


Figure S4: Van’t Hoff plots for the determination of thermodynamic data (ΔH and ΔS) for the interaction of the protease inhibitor, Ritonavir with HIV protease at different temperatures. (A) Wild Type (B) Mutant E35D↑G↑S (n = 3).

**References**

1. Pettersen, E.F., et al., *UCSF Chimera--a visualization system for exploratory research and analysis.* J Comput Chem, 2004. **25**(13): p. 1605-12.

2. Kožíšek, M., et al., *Characterisation of mutated proteinases derived from HIV-positive patients: enzyme activity, vitality and inhibition.* Collection of Czechoslovak chemical communications, 2004. **69**(3): p. 703-714.
